# Data on haplotype-supported immunoglobulin germline gene inference

**DOI:** 10.1016/j.dib.2017.06.031

**Published:** 2017-06-27

**Authors:** Ufuk Kirik, Lennart Greiff, Fredrik Levander, Mats Ohlin

**Affiliations:** a Dept. of Immunotechnology, Lund University, Lund, Sweden; b Dept. of Clinical Sciences, Division of Otorhinolaryngology, Head and Neck Cancer, Lund University, Sweden; c Dept. of Otorhinolaryngology, Skåne University Hospital, Lund, Sweden

**Keywords:** Antibody, Gene inference, Germline repertoire, Immunoglobulin germline gene, Transcriptome, Validation

## Abstract

Data that defines IGHV (immunoglobulin heavy chain variable) germline gene inference using sequences of IgM-encoding transcriptomes obtained by Illumina MiSeq sequencing technology are described. Such inference is used to establish personalized germline gene sets for in-depth antibody repertoire studies and to detect new antibody germline genes from widely available immunoglobulin-encoding transcriptome data sets. Specifically, the data has been used to validate (Parallel antibody germline gene and haplotype analyses support the validity of immunoglobulin germline gene inference and discovery (DOI: 10.1016/j.molimm.2017.03.012) (Kirik et al., 2017) [1]) the inference process. This was accomplished based on analysis of the inferred germline genes’ association to the donors’ different haplotypes as defined by their different, expressed IGHJ alleles and/or IGHD genes/alleles. The data is important for development of validated germline gene databases containing entries inferred from immunoglobulin-encoding transcriptome sequencing data sets, and for generation of valid, personalized antibody germline gene repertoires.

**Specifications Table**

**Table:** 

Subject area	*Biology, Medicine*
More specific subject area	*Immunobiology*
Type of data	*Sequence reads, tables, figures*
How data was acquired	*Next generation sequencing using Illumina MiSeq technology; analysis using immunoglobulin repertoire inference software*
Data format	*Raw data, analyzed data*
Experimental factors	*Data processing was performed using pRESTO, Change-O, TIgGER, IgDiscover, GIgGle*
Experimental features	*Immunoglobulin M heavy chain variable domain-encoding genes were amplified by RT-PCR, sequenced by next generation sequencing technology, and analyzed by bioinformatics approaches.*
Data accessibility	*FASTQ raw sequence data files are available from the European Nucleotide Archive, study accession number: PRJEB18926. Data is within this article. Code available at*https://github.com/ukirik/giggle

**Value of the data**

• The data is valuable for development of computational inference approaches that feature improved confidence in the outcomes of the inference process.
• The data is valuable for development of validated immunoglobulin germline gene databases.
• The data is valuable for validation of computational inference of personalized antibody germline gene repertoires.
• The data is valuable for the analytical process preceding studies of evolution of immune responses.

## 1 Data

**Fig. 1:**
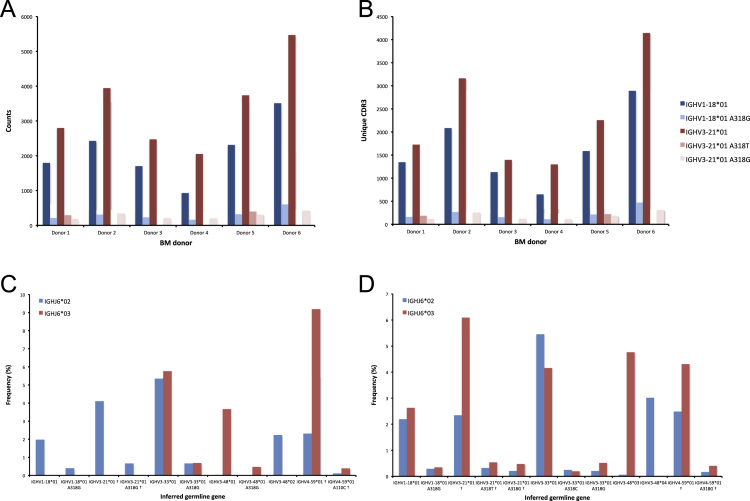
Germline gene variants of IGHV1-18 and IGHV3-21 inferred by IgDiscover when a starting germline database extending beyond codon 105 was used to initiate the process. The number of sequence counts (A) and unique CDRH3 (B) are shown. Examples (IGHV1-18, IGHV3-21, IGHV3-33, IGHV3-48, and IGHV4-59) of germline genes with new inferred variants, mostly in codon 106, and their similar association to the two different alleles of IGHJ6 of donor 4 (C) and donor 5 (D) are shown. Segregation of different established alleles of IGHV3-48 to the two alleles of IGHJ6 is also shown for comparison. † defines that the name of only one of a set of different alleles of the gene that cannot be differentiated by the analysis approach is shown.

**Fig. 2:**
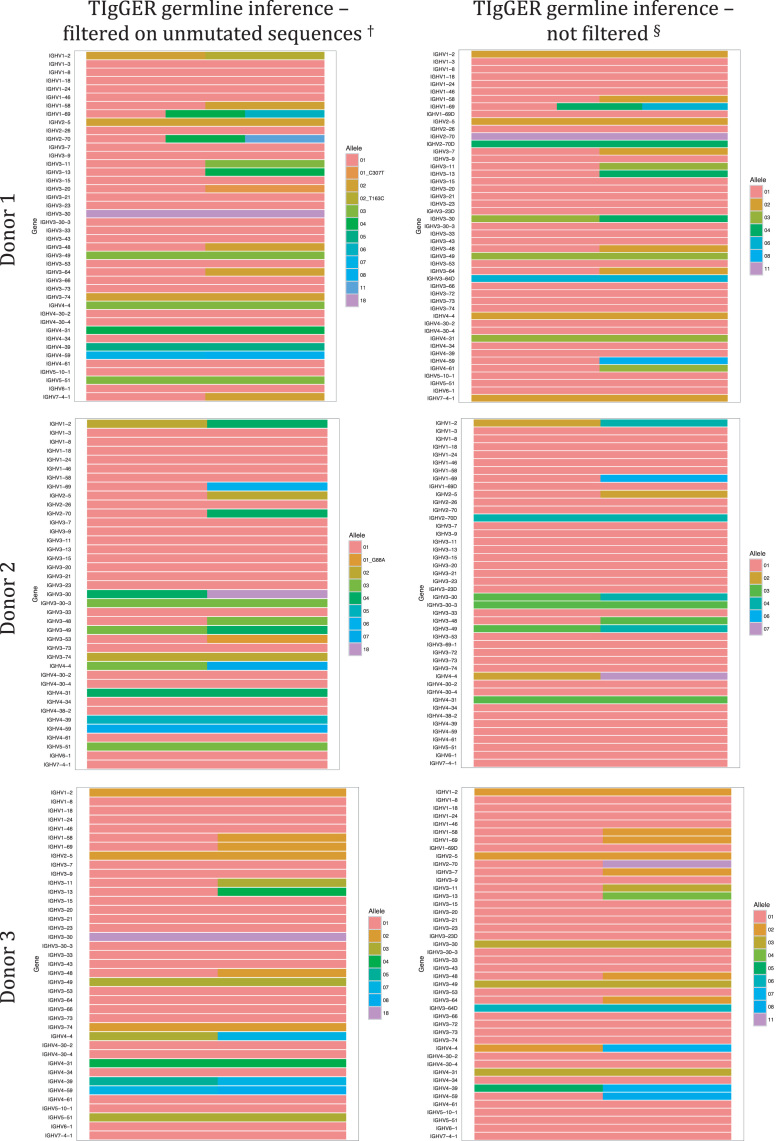

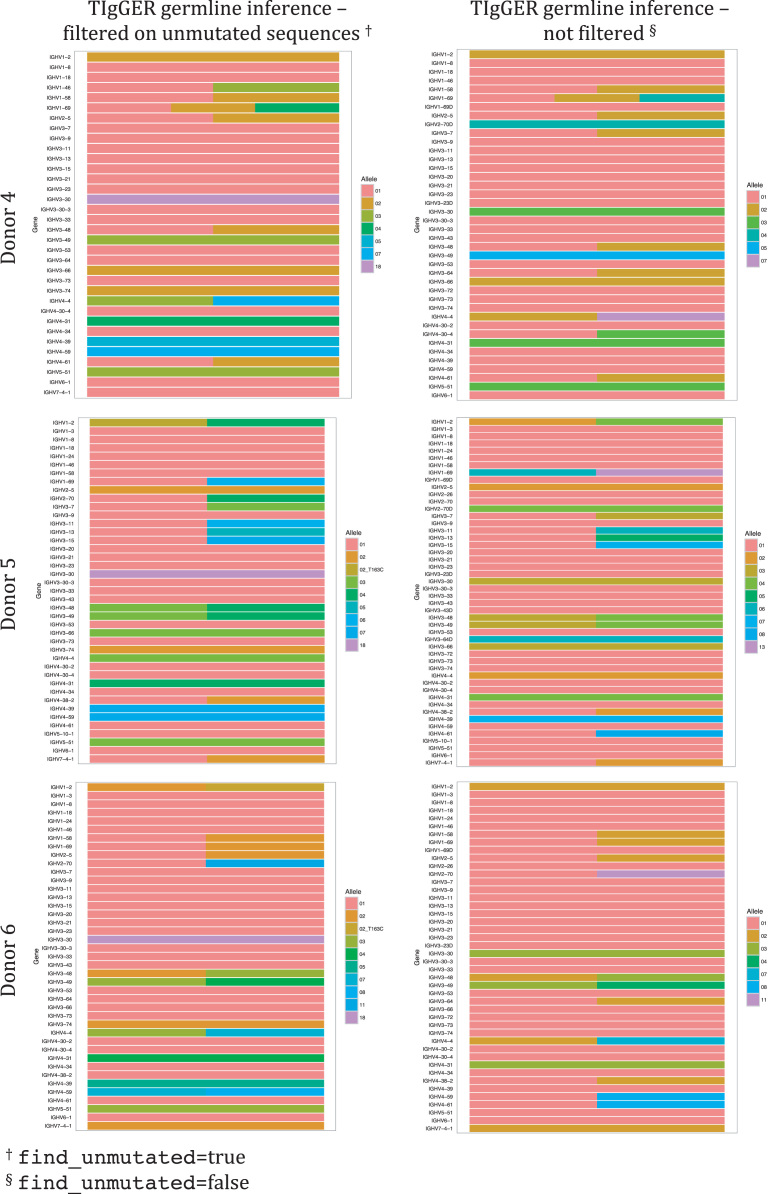
Genotype inferred by TIgGER using IgM-encoding transcripts of BM. Note difference in the calling of IGHV1-2. Heterozygous state of IGHV1-2 (*02/*p06) is inferred in subjects 1 and 6 only when argument find_unmutated=true while it is inferred in subject 2 (*02/*04) independently of the setting of find_unmutated. Heterozygous state of IGHV3-7 (*01/*02) is inferred in subjects 1, 3, and 4 only when argument find_unmutated=false while it is inferred in subject 5 (*01/*03) independently of the setting of find_unmutated. Heterozygous state of IGHV3-20 (*01/*01 C307T) is inferred in subject 1 only when argument find_unmutated=true and the allele variant is not at all inferred in donor 3. Heterozygous state of IGHV3-64 is inferred in donors 1, 3, 4, and 6 when argument find_unmutated=false and in donor 1 when argument find_unmutated=true. ,

**Fig. 3:**
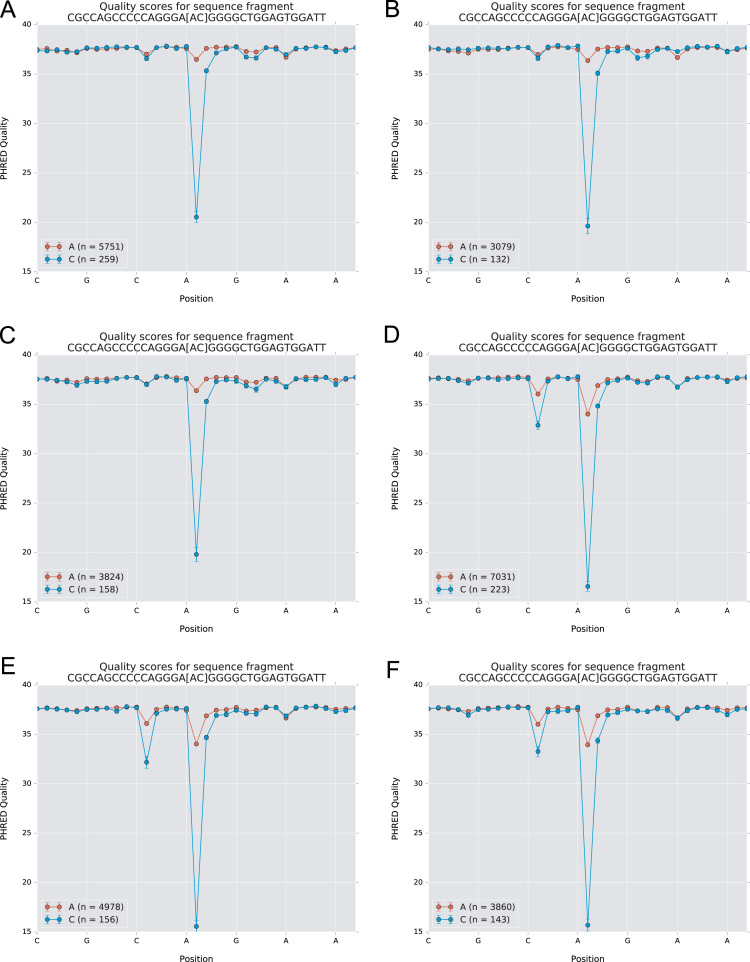

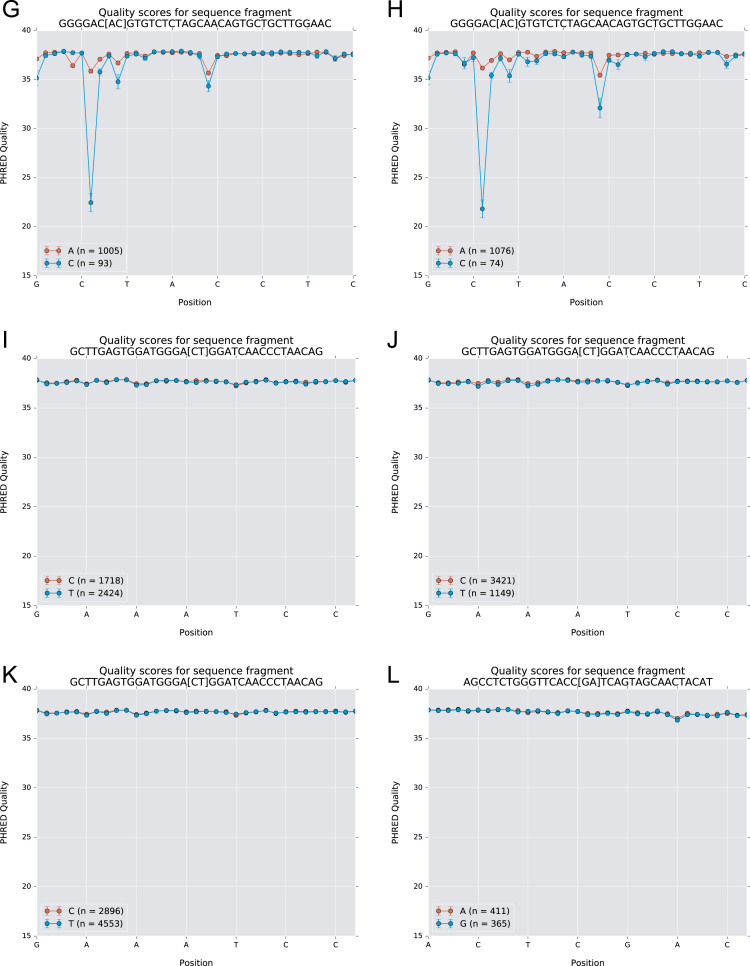

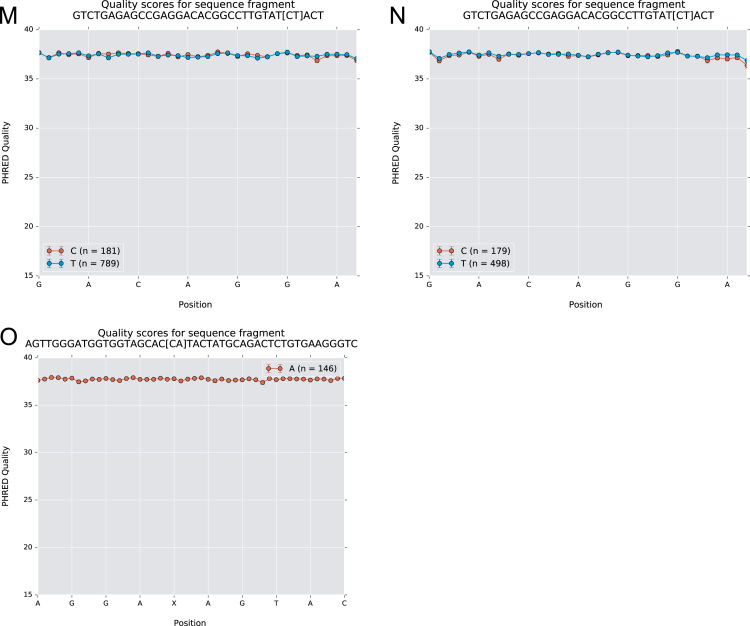
Quality score of sequencing reads representing germline genes inferred by IgDiscover. Sequence reads representing sequence variant A143C of IGHV4-39 show lower read quality (donors 1 (A), 2 (B), 3, (C), 4 (D), 5 (E), and 6 (F)) of the nucleotide representing the allele-differentiating base as opposed to reads defining the corresponding unmutated alleles (IGHV4-39*01 and *07). Similarly, inferred allele IGHV6-1 A85C shows low read quality of the allele-differentiating base (donor 5 (G), donor 6 (H)). Sequence reads representing parts of the sequences of alleles of IGHV1-2*02 and IGHV1-2*04 (represented by nucleotide T163) and IGHV1-2*p06 (C163) of donors 1 (I), 5 (J), and 6 (K) show highly similar read quality. Sequences representing IGHV3-53*01 and IGHV3-53*01 G88A of donor 2 (L), IGHV3-20*01 and IGHV3-20*01 C307T of donor 1 (M) and donor 3 (N), and IGHV3-43D*01 C195A of donor 6 (O) show high quality of the allele-differentiating base calls. The analysed sequence is shown above each graph and the allele-differentiating base is highlighted within square brackets.

**Fig. 4:**
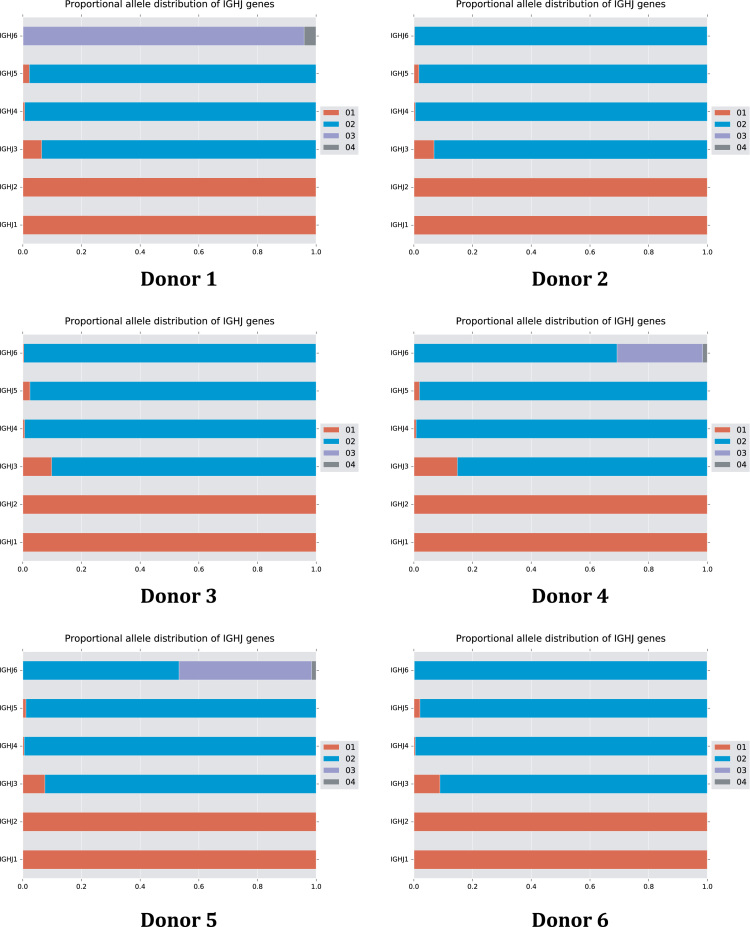
Perceived frequency of IGHJ gene usage in transcripts derived from donors 1–6, as analysed by IgDiscover.

**Fig. 5:**
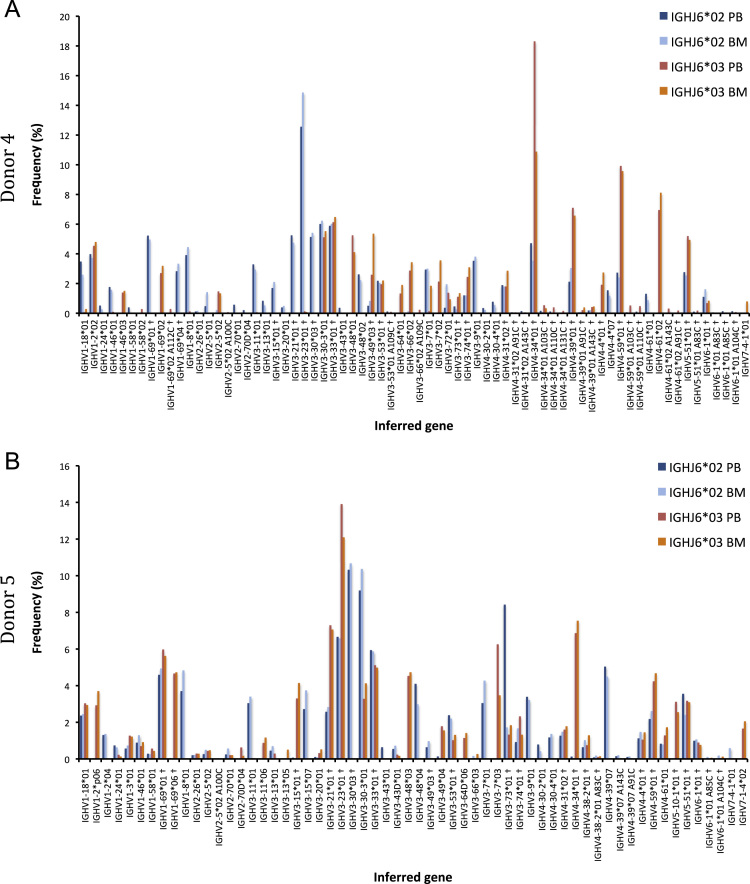
Summary of linkage of inferred germline genes/alleles of donors 4 (A) and 5 (B) to IGHJ6*02 and *03, as indicators of the donors’ two haplotypes, after analysis of transcripts found in bone marrow (BM) (also shown in Fig. 2 in Ref. [1]) and peripheral blood (PB). † defines that the name of only one of a set of different alleles of the gene that cannot be differentiated by the analysis approach is shown.

**Fig. 6:**
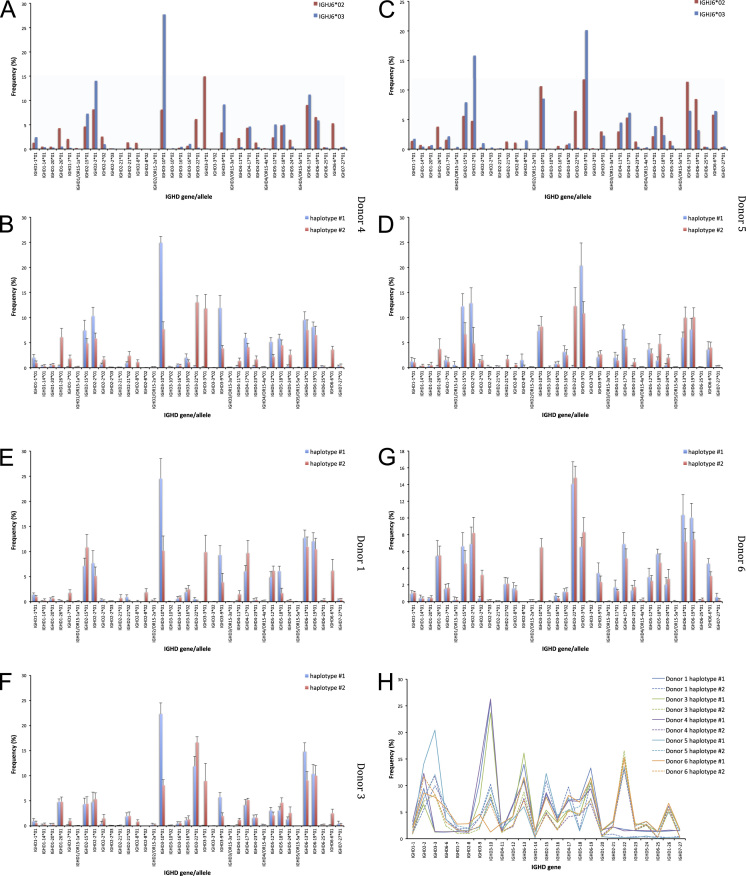
Association of IGHD gene expression IGHJ expression in unique IgM-encoding transcripts (at V_errors=0 and D_coverage>35 as defined by IgDiscover) derived from PB of donor 4 (A) and donor 5 (C). Association of IGHD gene expression (average±SD) to that of IGHV genes inferred as being present as two different alleles in transcripts derived from PB donors 1 (E), 3 (F), 4 (B), 5 (D), and 6 (G). A summary of IGHD gene usage (irrespective of allele call) based on association to expression of IGHV genes is shown (H).

**Fig. 7:**
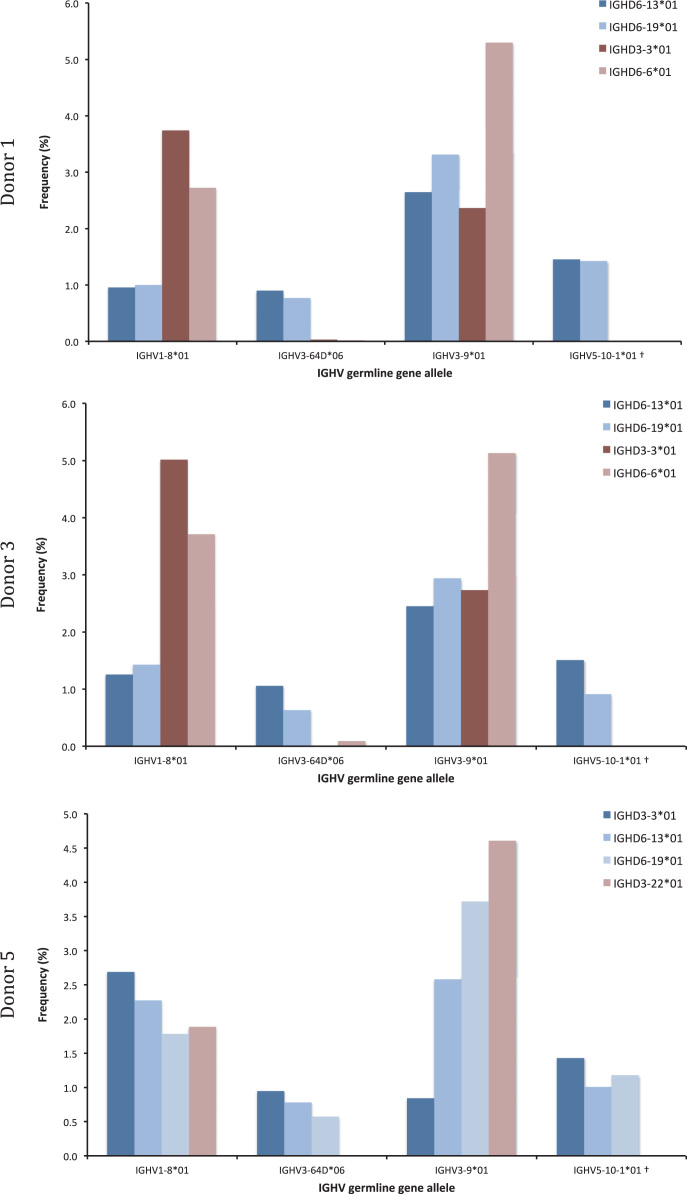
Linkage of IGHV1-8*01, IGHV3-64D*06, IGHV3-9*01, and IGHV5-10-1*01 to different IGHD genes in transcripts of donor 1, 3, and 5. While germline genes IGHV1-8*01 and IGHV3-9*01 were linked to the haplotype also carrying IGHD genes not present on both haplotypes, IGHV3-64D*06 and IGHV5-10-1*01 were not.

**Fig. 8:**
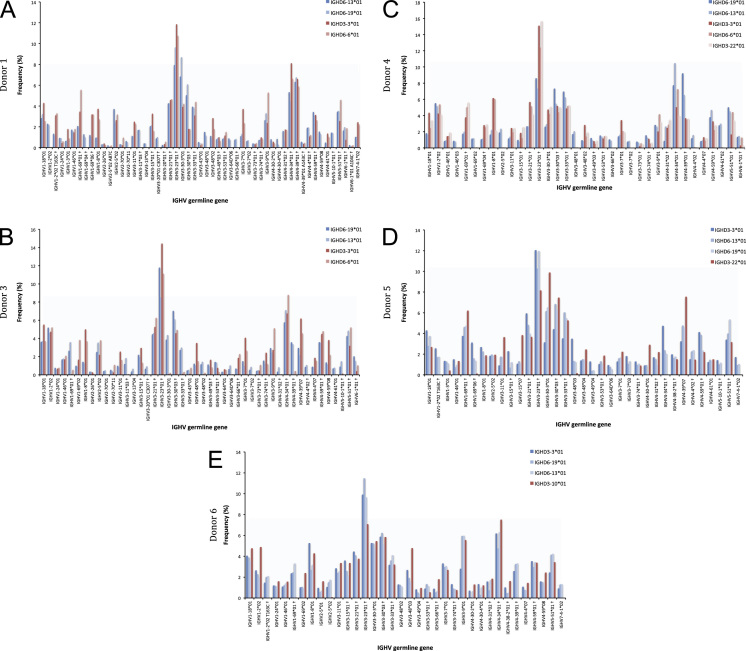
Association of IGHV genes/alleles of donors 1 (A), and 3–6 (B-E) with different IGHD genes as indicators of association with different haplotypes represented by IGHD. Analysis was performed on sequences found in cells of PB using the final filtered output of IgDiscover (diff=0). Only IGHV genes/alleles represented by at least 50 sequences with V_errors=0 and D_coverage>35 in the IGHD gene set shown in dark blue are shown. The frequencies of IGHV sequences associated to IGHD genes found in both haplotypes are shown in blue while the corresponding frequencies of IGHV sequences associated to IGHD genes expressed from only one of the inferred haplotypes are shown in red. † defines that the name of only one of a set of different alleles of the gene that cannot be differentiated by the analysis approach is shown.

**Fig. 9:**
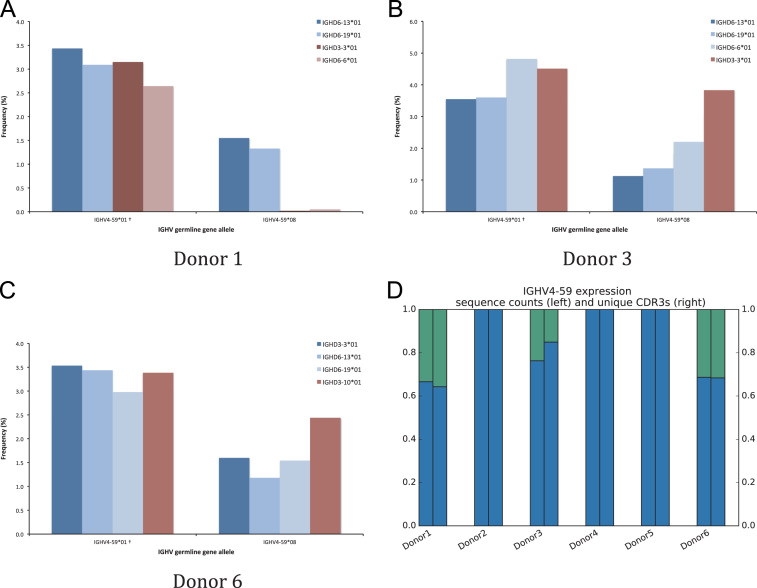
Differential association of inferred alleles of IGHV4-59 with different haplotypes of IGHD of donors 1 (A), 3 (B), and 6 (C). The frequencies of sequences associated to IGHD genes apparently expressed from both haplotypes are shown in blue while the frequencies of sequences associated to IGHD genes apparently expressed from only one of the haplotypes are shown in red. The fraction of reads represented by IGHV4-59*01 (blue) and *08 (green) in all three subjects is shown (fraction of sequences to the left and fraction of unique CDR3 to the right) (D). † defines that the name of only one of a set of different alleles of the gene that cannot be differentiated by the analysis approach is shown.

**Fig. 10:**
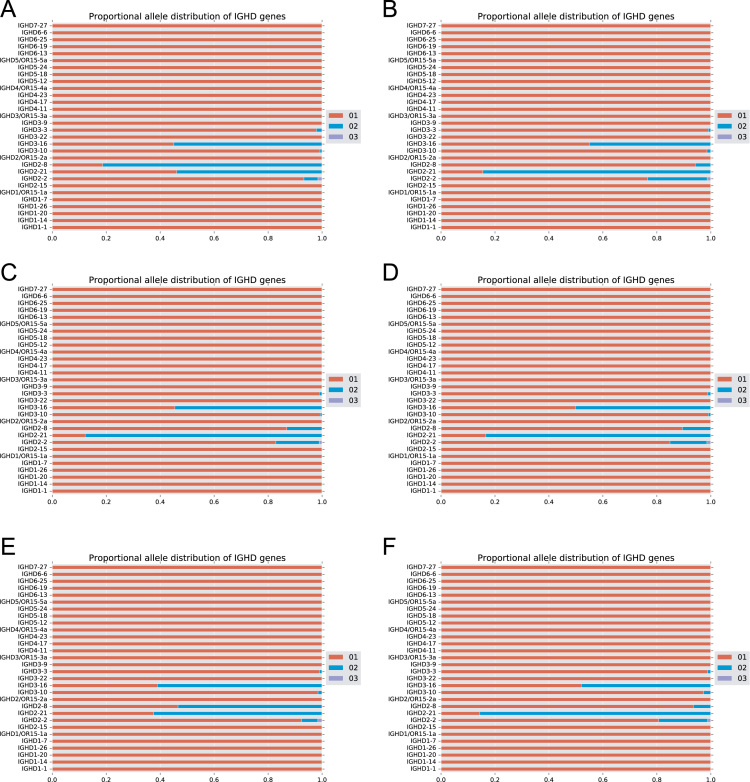
Apparent utilization of alleles of IGHD genes in IgM-encoding transcripts of BM of donors 1 (A), 2 (B), 3 (C), 4 (D), 5 (E), and 6 (F), as annotated by IgDiscover.

**Fig. 11:**
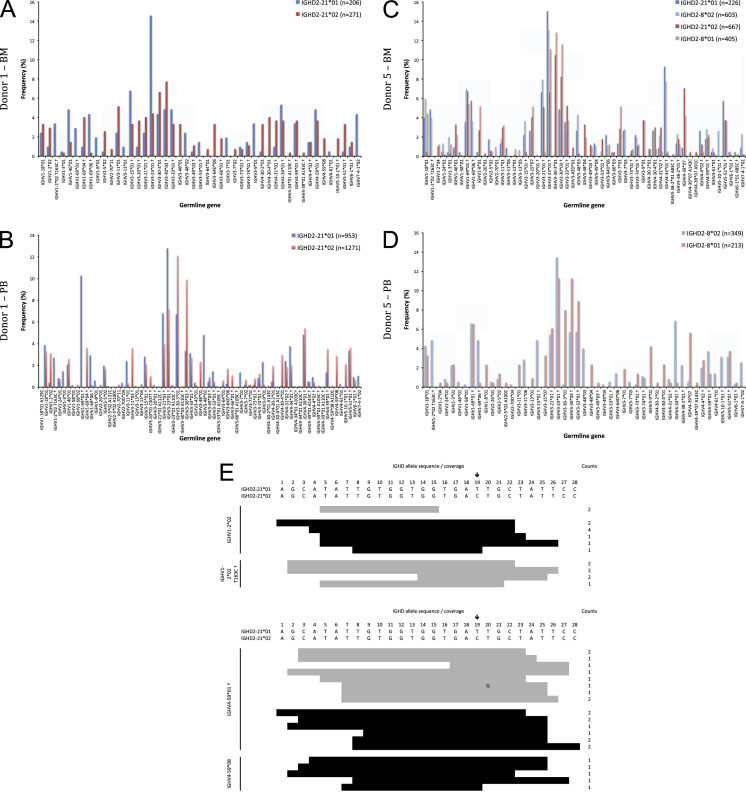
Immunoglobulin IGHV gene haplotype analysis based on heterozygous presence of IGHD alleles of donor 1 (A, B) and donor 5 (C, D). Transcripts found in BM (A, C) and PB (B, D) were analysed. The analysis of transcripts derived from PB employing IGHD2-21 was not included due to the low number of such sequences. Detailed sequence analysis (E) may be used to define whether or not IGHD allele assignments are appropriate. The rare association of reads of IGHV1-2*02 to IGHD2-21*01 (grey) instead of the expected IGHD2-21*02 (black) in some BM-derived transcripts of donor 1 (see A) does not cover the base within the IGHD that defines the individual alleles. IGHD2-21 allele calls for both alleles of IGHV4-59*01 include the allele-differentiating base, and rearrangements involving IGHV4-59*08 include the base identifying IGHD2-21*02. The arrow indicates the only base that differentiate IGHD2-21*01 and *02. Mutated bases within the sequences derived from IGHD genes are spelled out. † defines that the name of only one of a set of different alleles of the gene that cannot be differentiated by the analysis approach is shown.

The data of this article summarize the identity and accession numbers of sequencing data files (Table 1), the sizes of the sequence sets during the different stages of data processing (Table 2), and the outcome of validation of new inferred genes/alleles (Table 3), identified by use of IgDiscover and TIgGER. The frequencies of readily inferable [2] IGHD (Immunoglobulin heavy D-gene) genes used by the two haplotypes of five subjects are summarized (Table 4). Furthermore the data illustrate the effect of using a germline gene database that extends beyond codon 105 on gene inference (Fig. 1), and summarizes the outcome of TIgGER-based germline gene inference of six transcriptoms (Fig. 2). The data also illustrates how low sequencing quality scores are associated with some, but certainly not all, inferred germline gene alleles (Fig. 3), and summarizes IGHJ (Immunoglobulin heavy J-gene) alleles used by transcriptomes of six subjects (Fig. 4). The link between inferred IGHV (Immunoglobulin heavy V-gene) germline genes/alleles and different alleles of IGHJ6 in bone marrow (BM)- and peripheral blood (PB)-derived transcriptomes of two heterozygous subjects is shown (Fig. 5). The data summarizes linkage of different IGHD genes to two different haplotypes defined by alleles of IGHJ6 or defined by heterozygous IGHV genes (Fig. 6). The linkage of IGHV1-8, IGHV3-9, IGHV5-10-1, and IGHV3-64D germline genes to different haplotypes in subjects with two different IGHD gene-defined haplotypes (Fig. 7) is shown. Association of IGHV germline genes/alleles with particular IGHD genes in five subjects with different IGHD-defined haplotypes is shown (Fig. 8), as is the extent of association of alleles of IGHV4-59 to particular IGHD genes (Fig. 9). Finally, data describing assessment of alleles of IGHD genes detected in IgM-encoding transcriptomes of six subjects (Fig. 10), and of IGHV germline genes associated to the different alleles of IGHD genes in two subjects (Fig. 11) is shown.

**Table 1:** Summary of identity of sequenced samples of the study (European Nucleotide Archive (ENA) accession number PRJEB18926).^a^

Subject	Sample origin^b^	Replicate	Sequencing sample ID	Isotypes	ENA sample accession number	ENA experiment accession number
1	BM	1	P1882_1001	IgA, IgE, IgG, IgM	ERS1531209	ERX1875309
1	BM	2	P1882_1002	IgA, IgE, IgG, IgM	ERS1531210	ERX1875310
1	PB	1	P1882_1007	IgA, IgE, IgG, IgM	ERS1531215	ERX1875315
1	PB	2	P1882_1008	IgA, IgE, IgG, IgM	ERS1531216	ERX1875316
2	BM	1	P1882_1003	IgA, IgE, IgG, IgM	ERS1531211	ERX1875311
2	BM	2	P1882_1004	IgA, IgE, IgG, IgM	ERS1531212	ERX1875312
2	PB	1	P1882_1009	IgA, IgE, IgG, IgM	ERS1531217	ERX1875317
2	PB	2	P1882_1010	IgA, IgE, IgG, IgM	ERS1531218	ERX1875318
3	BM	1	P1882_1005	IgA, IgE, IgG, IgM	ERS1531213	ERX1875313
3	BM	2	P1882_1006	IgA, IgE, IgG, IgM	ERS1531214	ERX1875314
3	PB	1	P1882_1011	IgA, IgE, IgG, IgM	ERS1531219	ERX1875319
3	PB	2	P1882_1012	IgA, IgE, IgG, IgM	ERS1531220	ERX1875320
4	BM	1	P1882_1013	IgA, IgE, IgG, IgM	ERS1531221	ERX1875321
4	BM	2	P1882_1014	IgA, IgE, IgG, IgM	ERS1531222	ERX1875322
4	PB	1	P1882_1019	IgA, IgE, IgG, IgM	ERS1531227	ERX1875327
4	PB	2	P1882_1020	IgA, IgE, IgG, IgM	ERS1531228	ERX1875328
5	BM	1	P1882_1015	IgA, IgE, IgG, IgM	ERS1531223	ERX1875323
5	BM	2	P1882_1016	IgA, IgE, IgG, IgM	ERS1531224	ERX1875324
5	PB	1	P1882_1021	IgA, IgE, IgG, IgM	ERS1531229	ERX1875329
5	PB	2	P1882_1022	IgA, IgE, IgG, IgM	ERS1531230	ERX1875330
6	BM	1	P1882_1017	IgA, IgE, IgG, IgM	ERS1531225	ERX1875325
6	BM	2	P1882_1018	IgA, IgE, IgG, IgM	ERS1531226	ERX1875326
6	PB	1	P1882_1023	IgA, IgG, IgM^c^	ERS1531231	ERX1875331
6	PB	2	P1882_1024	IgA, IgG, IgM^c^	ERS1531232	ERX1875332

a Read numbers representing each sample/isotype are available in Supplementary Table EIV of Ref. [3].

b BM: bone marrow; PB: peripheral blood.

c No PCR product was derived using IgE-specific 3′-primers.

**Table 2:** Number of IgM-encoding sequences at different stages of the analysis process.

Donor	Tissue^a^	# of reads after filtering^b^	# of sequences after PRESTO pipeline^c^	# of unique sequences^d^	# of unique sequences with V_errors=0^d^	# of unique sequences with V_errors=0 & D_coverage>35%^d^
1	BM	258,988	261,967	86,135	47,233	43,006
	PB	nd	1,068,050	370,114	233,786	212,414
2	BM	194,555	197,949	90,181	58,685	52,815
	PB	nd	548,228	241,853	152,456	136,060
3	BM	278,426	281,711	70,515	28,827	26,400
	PB	nd	1,285,522	394,304	172,864	157,687
4	BM	339,935	345,021	91,511	45,510	40,850
	PB	nd	456,175	201,889	124,741	111,341
5	BM	318,207	324,269	106,047	63,924	57,998
	PB	nd	511,142	96,357	48,325	43,553
6	BM	406,893	412,689	152,125	85,956	77,603
	PB	nd	693,033	208,311	122,685	109,770

nd – not done.

a BM: bone marrow; PB peripheral blood

b Number of sequences used for initiation of the workflow towards TIgGER-based analysis.

c Number of sequences used for initiation of the workflow towards IgDiscover-based analysis.

d Number of unique sequences in the final filtered output obtained using IgDiscover as inference method

**Table 3:** Summary of sequence variants of germline genes not present in the IMGT germline gene database but inferred from BM transcript data using IgDiscover or TIgGER.

**Table 4:** Estimated frequency^*^ of use of readily identified IGHD germline genes [2] in haplotypes of five lymphocyte donors, and the ratio of estimated frequency^†^ of these genes in the two haplotypes.

## 2 Experimental design, materials and methods

IgM heavy chain variable domain-encoding gene repertoires were isolated by RT-PCR from transcriptomes of PB and BM collected out of season of most seasonal allergens from six allergic subjects [3]. Ethical approval and informed consent had been obtained from all donors. Sequencing was performed using the 2 × 300 bp MiSeq technology (Illumina, Inc., San Diego, CA, USA) at the National Genomics Infrastructure (SciLifeLab, Stockholm, Sweden) [3]. Details of sequence output and availability are outlined in Table 1. Data was pre-processed using pRESTO [4] and Change-O [5] as summarized in Fig. 1 in Ref. [1]. Germline gene inference was performed using TIgGER [6] and IgDiscover [7]. Additional bioinformatics analysis was performed as outlined elsewhere [1] including analysis performed using GIgGle (release 0.2) that is available under Apache License at https://github.com/ukirik/giggle. Immunoglobulin gene names and sequence numbering complies with the nomenclature defined by the International ImMunoGeneTics information system® (IMGT) (http://www.imgt.org) [8], [9].
